# A memory-efficient algorithm to obtain splicing graphs and *de novo* expression estimates from de Bruijn graphs of RNA-Seq data

**DOI:** 10.1186/1471-2164-15-S5-S6

**Published:** 2014-07-14

**Authors:** Sing-Hoi Sze, Aaron M Tarone

**Affiliations:** 1 Department of Computer Science and Engineering, Texas A&M University, College Station, TX 77843, USA; 2 Department of Biochemistry & Biophysics, Texas A&M University, College Station, TX 77843, USA; 3 Department of Entomology, Texas A&M University, College Station, TX 77843, USA

## Abstract

**Background:**

The recent advance of high-throughput sequencing makes it feasible to study entire transcriptomes through the application of *de novo *sequence assembly algorithms. While a popular strategy is to first construct an intermediate de Bruijn graph structure to represent the transcriptome, an additional step is needed to construct predicted transcripts from the graph.

**Results:**

Since the de Bruijn graph contains all branching possibilities, we develop a memory-efficient algorithm to recover alternative splicing information and library-specific expression information directly from the graph without prior genomic knowledge. We implement the algorithm as a postprocessing module of the Velvet assembler. We validate our algorithm by simulating the transcriptome assembly of *Drosophila *using its known genome, and by performing *Drosophila *transcriptome assembly using publicly available RNA-Seq libraries. Under a range of conditions, our algorithm recovers sequences and alternative splicing junctions with higher specificity than Oases or Trans-ABySS.

**Conclusions:**

Since our postprocessing algorithm does not consume as much memory as Velvet and is less memory-intensive than Oases, it allows biologists to assemble large libraries with limited computational resources. Our algorithm has been applied to perform transcriptome assembly of the non-model blow fly *Lucilia sericata *that was reported in a previous article, which shows that the assembly is of high quality and it facilitates comparison of the *Lucilia sericata *transcriptome to *Drosophila *and two mosquitoes, prediction and experimental validation of alternative splicing, investigation of differential expression among various developmental stages, and identification of transposable elements.

## Background

With the advance of high-throughput sequencing techniques, it is feasible to study entire transcriptomes through the application of *de novo *sequence assembly algorithms [1-8]. A popular strategy of transcriptome assembly algorithms is to first obtain a de Bruijn graph that contains all branching possibilities [7-10]. An additional step is then performed to construct predicted transcripts from the graph. This strategy is employed by Oases [10] and Trans-ABySS [9], which use output from Velvet [5] and ABySS [6] respectively to obtain predicted transcripts. One drawback of the approach is that Oases can be more memory-intensive than Velvet, which limits its application when computational resources are limited. Alternatively, Trinity [8] uses a different approach of first clustering the data, then constructing an individual de Bruijn graph for each cluster that has simple structure.

**Figure 1:**
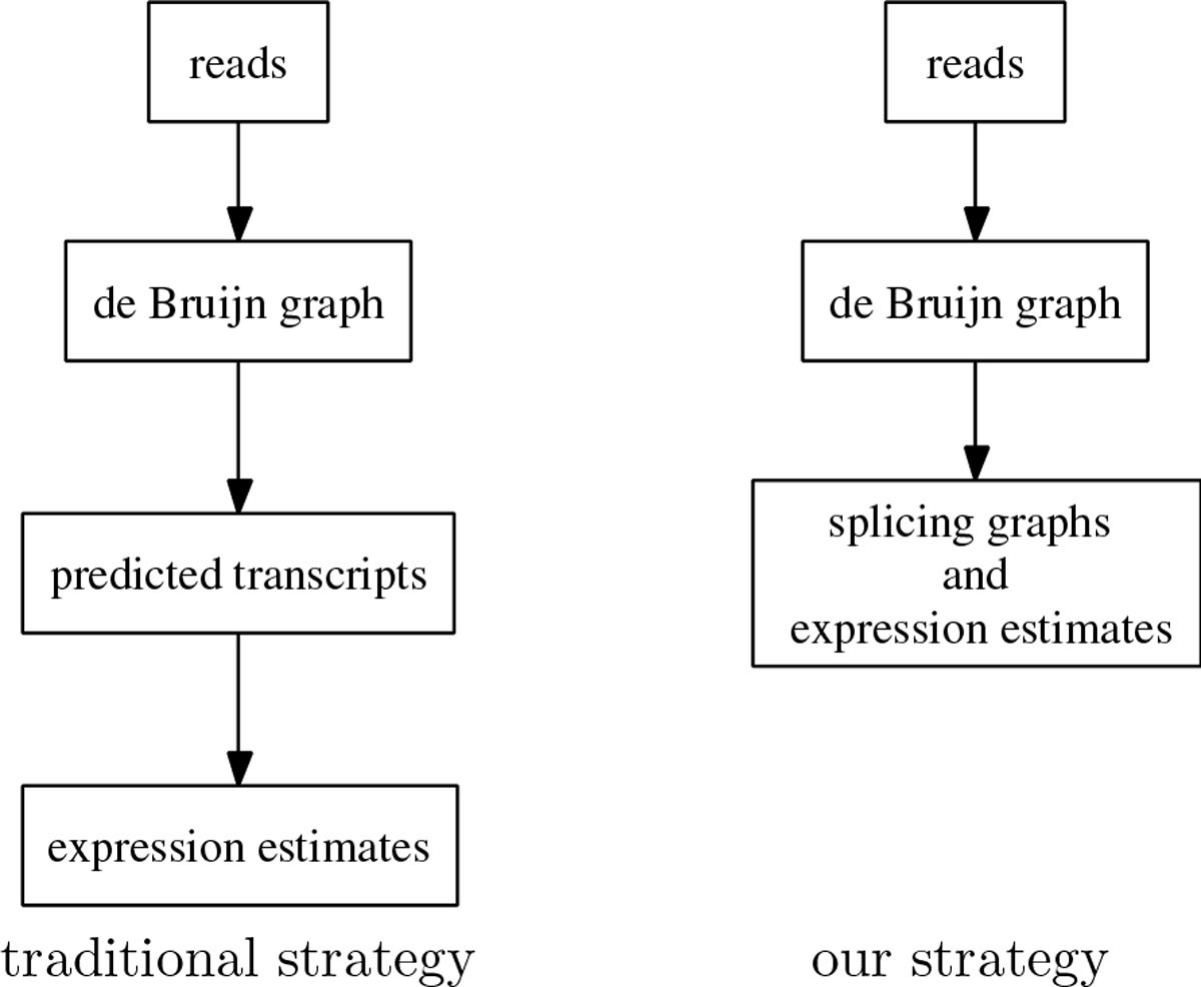
**Difference between traditional strategy and our strategy**.

We observe that it is possible to develop a memory-efficient algorithm to recover alternative splicing information directly from the intermediate de Bruijn graph structure that contains all branching possibilities (see Figure 1). Although many of the simpler components of the de Bruijn graph can already represent alternatively spliced variants of individual genes, the graph still contains big tangles that need to be addressed. We develop an algorithm to remove the complicated cycles in the de Bruijn graph, and extract acyclic components so that each of them represents a gene and its isoforms in almost all cases. Our goal is to preserve the alternative splicing information that is inherent within the reads as much as possible, and report these components as splicing graphs.

We implement the algorithm as a postprocessing module of Velvet. We validate our algorithm by simulating the transcriptome assembly of *Drosophila *using its known complete genome under the condition that all gene transcripts have high expression levels, and by performing *Drosophila *transcriptome assembly using publicly available RNA-Seq libraries. We further employ a *de novo *expression estimate to simultaneously evaluate the differential expression levels across libraries without requiring any prior knowledge of the genome, which was validated in [11]. We have applied our algorithm to perform transcriptome assembly of the non-model blow fly *Lucilia sericata *in [11].

## Methods

### De Bruijn graph

Given a set of reads and a parameter *k*, a de Bruijn graph is defined by constructing a vertex for each *k*-mer that appears within the reads. A pair of *k*-mers are connected by a directed edge if the (*k *− 1)-suffix of the first *k*-mer is the same as the (*k *− 1)-prefix of the second *k*-mer. It has been observed that the de Bruijn graph can be used to implicitly assemble these reads through linking together the same *k*-mer that appears in different reads [12,13]. Since the number of vertices and edges in a de Bruijn graph depends on the number of distinct *k*-mers from the reads rather than the total number of reads, this strategy is very popular among short read assembly algorithms for high-throughput sequencing data [2,3,5-7].

### Postprocessing algorithm

In order to retain alternative splicing information, Heber et al [14] developed an EST assembly algorithm that retains all the junctions in the de Bruijn graph. By imposing a *k*-mer coverage cutoff, each component becomes a splicing graph that specifies the alternatively spliced variants of a gene. While this strategy was proved to be successful for EST assembly, there are significant challenges in transcriptome assembly from high-throughput sequencing data that are caused by the shorter reads.

We develop a postprocessing algorithm that extracts the de Bruijn graph from Velvet [5] to construct non-linear splicing graphs that represent the transcriptome. In order to retain as much alternative path information as possible, Velvet is applied without using the tour bus algorithm that removes the bubbles in the graph, while still allowing the removal of short tips. Each node returned from Velvet corresponds to a maximal succession of vertices with no branches.

### SNPs

**Figure 2:**
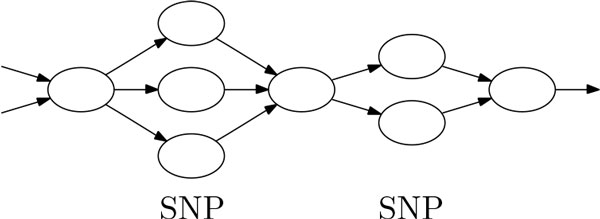
**Illustration of two successive sets of nodes that contain SNPs**. The sequences within all the nodes in the second column and the sequences within all the nodes on the fourth column must be of the same length in order to contain SNPs. Note that there can be more than one SNP within each of these columns, and all these nodes will be merged into a single node. Other incoming edges that go into the starting node and other outgoing edges that go out of the final node are allowed.

In order to remove SNPs that are not related to alternative splicing but will create branches in the graph, we search for the following structure: starting from a node, consider the nodes at the end of all its outgoing edges. If the sequences associated with all these nodes are of the same length with long enough matches and very few mismatches, each of these nodes has exactly one outgoing edge that all go to the same final node, there are no other branches going into or out of any of these nodes and no other branches going into the final node, and the structure does not contain a forward node and the corresponding backward node at the same time, we think of all the mismatches within this split-then-merge structure as SNPs. We repeat the procedure at the final node to look for successive split-then-merge structures, and merge all the nodes involved into a single node (see Figure 2 for an example). Note that this strategy only merges together obvious SNPs, and it does not resolve short indels.

### Strongly connected components

**Figure 3:**
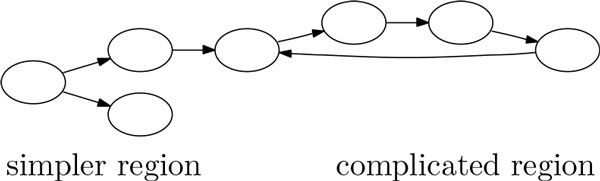
**Example of the decomposition of a connected component**. Each of the three edges on the left-hand side is a strongly connected component by itself, and the subgraph containing these three edges represents the simpler region. The cycle on the right-hand side is one single strongly connected component that represents the complicated region.

We observe that connected regions within the de Bruijn graph that are relatively free of cycles are likely to belong to the same gene. We decompose each connected component into a collection of edge-disjoint strongly connected components, with each strongly connected component being either just a single edge or a maximal subgraph with each vertex reachable from any other vertex. The regions within a strongly connected component that is not just a single edge represent the complicated regions that must contain a cycle, while the remaining regions represent the simpler regions that contain no cycles (see Figure 3 for an example). This step can be performed by depth-first search with running time that is proportional to the size of the graph [15].

### Forward-backward tangles

Since Velvet assembles the forward and the backward strands simultaneously, each gene should be represented by two disjoint components, one on each strand, which do not contain any cycles. Although there are no more cycles after removing the strongly connected components that are not just a single edge, it is still possible to have forward-backward tangles in which a forward node and the corresponding backward node reside within the same connected component. These forward-backward tangles can be identified by depth-first search [15].

### Splicing graphs

We extract all the nodes within the strongly connected components that are not just a single edge and within the forward-backward tangles. We treat each node as an individual assembly that consists only of a single node while ignoring the junction information within these complicated regions. We then remove these nodes and their adjacent edges, and extract the connected components in the remaining graph. Each of these connected components does not contain cycles and should mostly represent alternatively spliced variants of one gene. Only one of the two possible orientations is retained for each extracted node and each connected component.

### Junction adjustment

**Figure 4:**
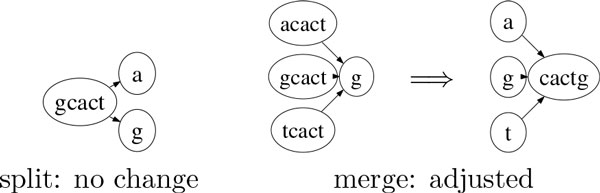
**Example of junction adjustment**.

Since adjacent vertices in a de Bruijn graph share overlapping sequence fragments of length *k *− 1 according to the definition of a de Bruijn graph, the location of junctions is imprecise and this representation is hard to interpret. Although the overlaps can be eliminated by following the strategy in [5] to remove the first *k *− 1 letters of the sequence in each node, the location of junctions is still imprecise and the beginning part of some of the sequences is missing. To resolve these uncertainties, we start with the non-overlapping strategy employed in [5] and consider two cases: at a split junction in which a path branches in more than one direction, the junction is precise and no change is necessary; at a merge junction in which more than one path meets at a node, we move the maximum number of shared letters in the suffix part of these paths to the meeting node to make the junction precise (see Figure 4 for an example).

Note that there can still be ambiguities due to the presence of repeating patterns across junctions. Since the graph no longer contains cycles after the previous processing steps, we recover the first *k *− 1 letters in each starting node with no incoming edges by restoring the removed letters. After these adjustments, we consider each resulting component as a splicing graph that specifies the alternative splicing paths of one gene. Note that we only resolve simple cases and do nothing when there are simultaneously a split and a merge at the two ends of an edge. To remove very short assemblies, we retain only the splicing graphs in which all paths from a source to a sink have sequence length above 2*k *− 1.

### *De novo* expression estimate

In order to evaluate differential expression levels in a non-model organism in which no prior information is available, we employ a measure of number of reads per kilobase of node per million reads (RPKM) [11] that is similar to the statistics used by [16] and [17]. Since there is no information about exons in a *de novo *assembly, reads that appear in the assembly are used instead of mapped reads. Also, each node in a splicing graph is evaluated instead of each exon, with each read that contains a *k*-mer within a node contributing to that node. Within each node, a RPKM estimate is reported independently for each library within the same assembly. A validation of the *de novo *RPKM values was given in [11] that shows strong correlations of these values with the ones given by Cufflinks [18] on genes without alternative splicing and good correlations on nodes from genes with alternatively splicing.

### Postprocessing software

A software program implementing our postprocessing algorithm is available at http://faculty.cse.tamu.edu/shsze/postprocess. In order to make the results directly applicable to other software during downstream analysis, we represent each assembly in an annotated FASTA format, in which each potentially non-linear structure is represented by a collection of nodes, with connecting edge information and RPKM values for each library embedded within the name of each node.

## Results and discussion

### *Drosophila melanogaster* simulations

To simulate the transcriptome assembly of *Drosophila*, we extracted all gene transcripts from the *D*. *melanogaster *genome. For each gene transcript, we randomly pick reads until an average nucleotide coverage of 100 is reached while allowing varying percentages of mismatches in the reads, giving 70598749 reads of length 75.

We applied Velvet by setting the parameters max_branch_length, max_divergence and max_gap_count to 0, while enabling read_trkg. We performed assemblies over different values of hash length *k *and cov_cutoff *c*. We extracted the de Bruijn graph from the LastGraph file and applied our postprocessing algorithm. Since *de novo *sequence assembly is performed mostly on non-model organisms and possible function of the assembled sequences is accessed with respect to a closely related organism, we used translated BLAST search [19] to simulate its usage.

While it is possible to recover 90% of the *Drosophila *genes under ideal conditions when there are no mismatches in the reads [11], Table 1 shows that the assemblies were still of high quality for 0.1% mismatches, with more than 73% of genes recovered. Table 2 shows that the performance for 0.2% mismatches was much worse, with only about half of the genes recovered. When *k *is small, the larger number of nodes in the simulated *Drosophila *assemblies can contain more information, although a larger proportion of them were in tangles and they were more likely to be in complicated regions. The extraction of strongly connected components reduced the size of the most complicated region by about half.

**Table 1:** Statistics of the simulated transcriptome assemblies of *Drosophila* using its known complete genome over different values of *k *and *k*-mer coverage cutoff *c *with 0.1% mismatches in the reads.

*k_c*	initial nodes	largest tangle	largest SCC	splicing graphs	max length	N50	>1-node graphs	max nodes	avg nodes	SNPs	total hits	unique hits	>1-hit graphs	max hits	time (mins)	memory (GB)
25_3	38884	17900	9937	15713	37380	2366	1361	3106	10	883	12731	10162	643	27	80,3	21,2
25_5	34822	16979	9255	15521	37380	2374	1351	266	7	517	12708	10160	643	27	80,3	21,2
25_10	34494	16712	9057	15486	37380	2373	1345	194	7	481	12699	10158	639	27	80,3	21,2

31_3	28342	5037	2080	13819	45158	2704	1719	1007	7	496	12523	11112	546	12	76,3	18,2
31_5	27307	4971	1898	13740	45158	2714	1717	167	6	381	12494	11110	552	13	76,3	18,2
31_10	27265	4947	1885	13829	45158	2704	1698	161	6	377	12536	11109	542	13	76,3	18,2

Initial nodes denotes the number of nodes that are in the initial assembly. Largest tangle denotes the number of nodes of the largest connected component. Largest SCC denotes the number of nodes of the largest strongly connected component. Splicing graphs denotes the number of splicing graphs. Max length denotes the length (in nucleotides) of the longest path over all splicing graphs. N50 denotes the N50 value of the length (in nucleotides) of the longest path in each graph. >1-node graphs denotes the number of graphs with more than one node. Max nodes denotes the maximum number of nodes in these non-linear graphs. Avg nodes denotes the average number of nodes in these non-linear graphs. SNPs denotes the number of SNPs recovered. Total hits denotes the total number of hits from translated BLAST search of each node to *Drosophila *(isoforms are considered the same gene, only the top hit with *E*-value below 10^−^^7 ^is included for each node in a splicing graph, and hits from nodes within the same splicing graph to the same gene are counted once). Unique hits denotes the number of unique hits to different genes. >1-hit graphs denotes the number of splicing graphs that have BLAST hits to more than one gene. Max hits denotes the maximum number of different genes that have BLAST hits to a splicing graph. Time (mins) denotes the computational time in minutes, with the values to the left and to the right of "," indicating the running time of Velvet and our postprocessing algorithm respectively. Memory (GB) denotes the memory requirement in gigabytes, with the values to the left and to the right of "," indicating the memory requirement of Velvet and our postprocessing algorithm respectively.

**Table 2:** Statistics of the simulated transcriptome assemblies of *Drosophila* using its known complete genome over different values of *k *and *k*-mer coverage cutoff *c *with 0.2% mismatches in the reads. The notations are the same as in Table 1.

*k_c*	initial nodes	largest tangle	largest SCC	splicing graphs	max length	N50	>1-node graphs	max nodes	avg nodes	SNPs	total hits	unique hits	>1-hit graphs	max hits	time (mins)	memory (GB)
25_3	45305	23504	15883	13240	26909	2255	634	8671	27	2049	8258	6188	315	16	94,3	30,2
25_5	29090	16349	11411	11734	27251	2321	606	1832	11	337	8156	6180	321	12	94,3	30,2
25_10	26297	15235	10367	11606	27251	2329	595	165	8	257	8116	6176	319	13	94,3	30,2

31_3	23544	5604	2331	11993	44990	2536	583	1520	12	611	9561	8488	281	17	83,3	21,2
31_5	19869	4299	2097	11650	44990	2545	571	253	7	248	9548	8488	281	13	83,3	21,2
31_10	19541	4222	2056	11642	44990	2545	572	96	7	233	9544	8484	281	13	83,3	21,2

When *k *is small, the larger number of splicing graphs resulted in more complete assemblies, although the sequences were shorter and thus more fragmented. When *k *is large, the maximum and median (N50) lengths of splicing graphs approached the maximum and median lengths of gene transcripts in the known *Drosophila *genome, which are 69439 and 3231 respectively. Between 5 to 12% of splicing graphs had non-linear structures. These values are a significant portion of the percentage of known *Drosophila *genes that have more than one alternatively spliced variant, which is 27%. A small number of SNPs were recovered, which may be due to variations in repeats or the inability to separate gene families. 

When compared to the total number of BLAST hits, the number of unique BLAST hits to different *Drosophila *genes was not much smaller. When compared to the total number of splicing graphs, only a small number of graphs have BLAST hits to more than one gene. Within these graphs, the maximum number of different genes that have BLAST hits to a graph was small, thus we have mostly achieved the goal that each splicing graph should represent alternatively spliced variants of only one gene. When the *k*-mer coverage cutoff *c *is 3, the number of junctions and some of the splicing graphs were very large. Otherwise, the results were similar over different cutoffs for the same value of *k*. This is due to the consistent high coverage that is guaranteed by the simulation.

**Figure 5:**
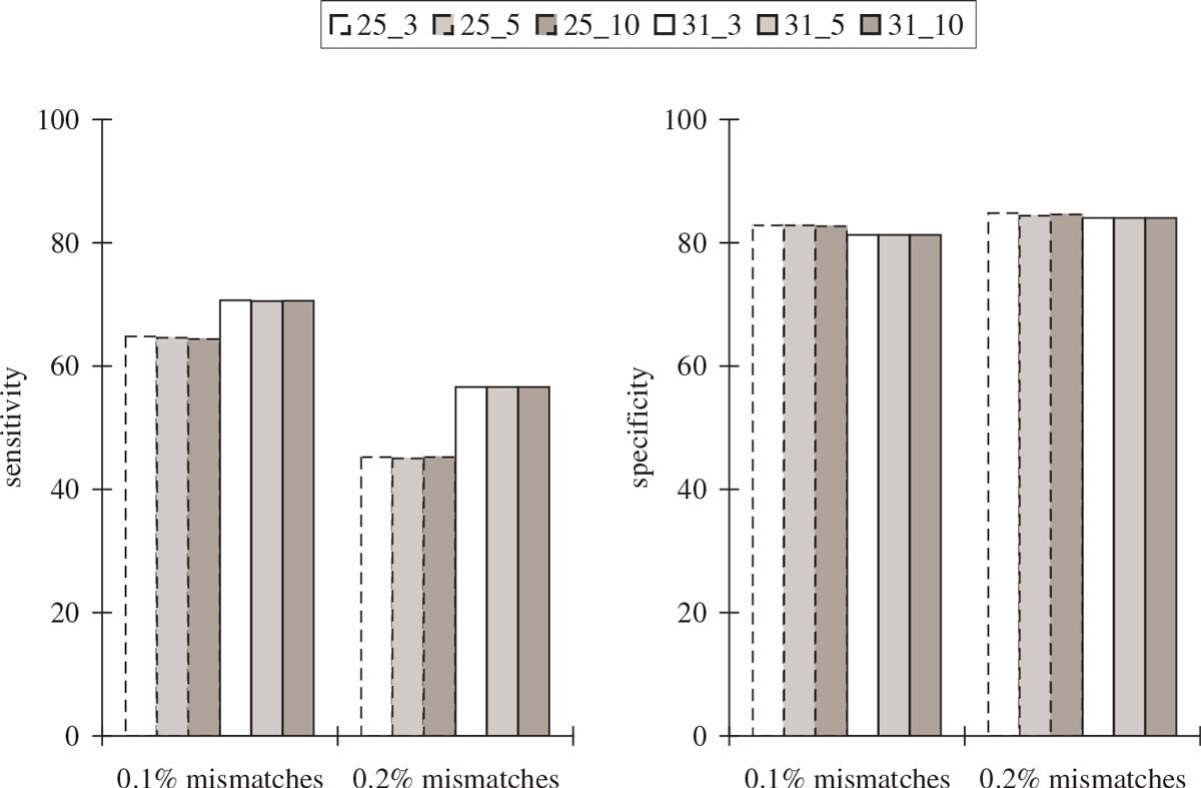
**Comparisons of the protein sequence BLAST results in the simulated transcriptome assemblies of *Drosophila* using its known complete genome over different values of *k *and *k*-mer coverage cutoff *c *(represented by *k_c*) with varying percentages of mismatches in the reads**. Sensitivity is defined to be the percentage of coding positions in the genome that are recovered in the assembly considering only *Drosophila *gene transcripts that are found in BLAST hits (each position that is within some coding region is counted once). Specificity is defined to be the percentage of predicted transcript positions in the assembly that are included in BLAST alignments considering only predictions that have BLAST hits.

**Figure 6:**
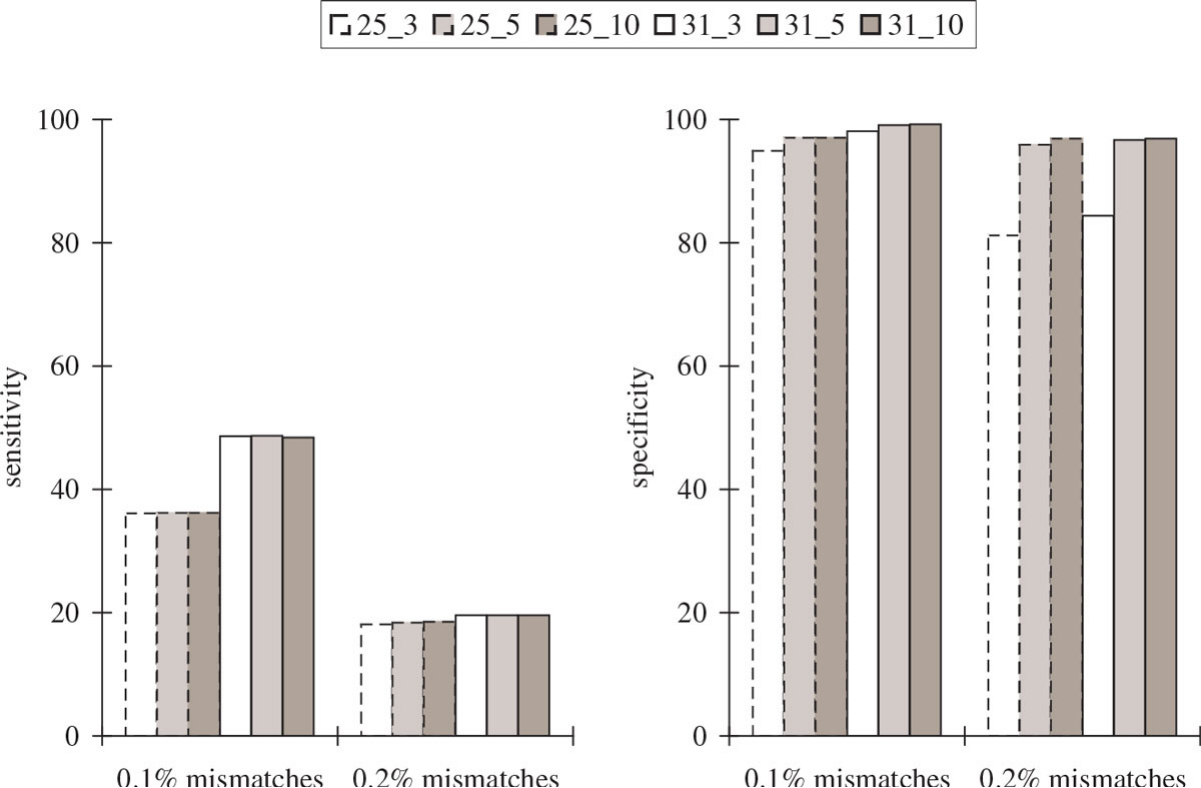
**Comparisons of the alternative splicing junction results in the simulated transcriptome assemblies of *Drosophila* using its known complete genome over different values of *k *and *k*-mer coverage cutoff *c *(represented by *k_c*) with varying percentages of mismatches in the reads**. Sensitivity is defined to be the percentage of junctions in the gene transcripts of *Drosophila *that appear somewhere in the assembly. Specificity is defined to be the percentage of junctions in the assembly that appear somewhere in the gene transcripts of *Drosophila*. Junctions in the gene transcripts of *Drosophila *are defined by concatenating the two sequences of length *k *that are immediately to the left and immediately to the right of all alternative splicing locations to obtain a sequence of length 2*k*. Junctions in the assembly are defined by concatenating the two *k*-mers at the beginning and ending nodes of an edge to obtain a sequence of length 2*k *after the elimination of overlapping sequence fragments between adjacent nodes. Up to three mismatches are allowed when looking for these sequence occurrences.

Figure 5 and Figure 6 show that while the sensitivity with respect to protein sequence BLAST and alternative splicing junctions decreases as the percentage of mismatches increases, the specificity remained high. For 0.2% mismatches, only about half of the coding positions and about 20% of the alternative splicing junctions were recovered, indicating that it is much harder to recover the alternative splicing junctions.

### *Drosophila* public libraries

To investigate the transcriptome assembly of *Drosophila *under realistic conditions, we obtained reads of length 75 from six RNA-Seq libraries in [20] at the sequence read archive [21] that include the following developmental stages: 2-16 hours embryos (SRX019647), third instar larvae (SRX019648), mixed pupae (SRX019651, two replicates), adult females (SRX019652), and adult males (SRX019653). Since sequence quality decreases toward the end of a read, we trimmed each read by removing all positions including and to the right of the first position that has a quality score of less than 15, giving a total of 102262392 reads with average length 40. We compare the performance of our postprocessing algorithm to Oases and Trans-ABySS on machines with 32 GB physical memory. Since the memory requirement of Oases exceeds 32 GB when the *k*-mer length is small, we fix *k *to 35.

For Oases, Velvet was applied with hash length *k *without setting cov_cutoff while enabling read_trkg. Oases was then applied on the results from Velvet with cov_cutoff *c*. For Trans-ABySS, abyss-pe was applied with *k*-mer size *k*, mean *k*-mer coverage threshold c, and minimum number of pairs *n*=10. Trans-ABySS was then applied on the results from abyss-pe by utilizing the assembly.py script with the single *k*-mer length. For our postprocessing algorithm and Oases, all reads were treated as single-end reads.

Table 3 shows that the maximum and median lengths of the assemblies were much smaller for all the three algorithms when compared to the simulation results. The number of unique BLAST hits decreases as the *k*-mer coverage cutoff *c *increases, while the ratio of the total number of BLAST hits to the number of number of unique BLAST hits was between 1.5 to 4, indicating sequence fragmentation. Trans-ABySS had the longest assemblies, while Oases had the largest number of unique BLAST hits. Although our postprocessing algorithm had the shortest assemblies, it had a larger number of unique BLAST hits than Trans-ABySS when the *k*-mer coverage cutoff *c *is 3.

**Table 3:** Comparisons of the *Drosophila* transcriptome assemblies of our postprocessing algorithm, Oases and Trans-ABySS using six publicly available libraries over different values of *k*-mer coverage cutoff *c*.

postprocess																
***k_c***	**initial nodes**	**largest tangle**	**largest SCC**	**splicing graphs**	**max length**	**N50**	**>1-node graphs**	**max nodes**	**avg nodes**	**SNPs**	**total hits**	**unique hits**	**>1-hit graphs**	**max hits**	**time (mins)**	**memory (GB)**

35_3	227614	178545	88094	75367	10539	544	2048	124	6	16703	38448	10719	392	5	86,18	22,2
35_5	125414	87895	41654	47958	8678	705	1720	93	6	11334	27010	9889	429	13	86,17	22,2
35_10	57978	31785	12695	27695	6383	705	1020	63	6	5034	17271	8070	308	5	86,16	22,2

**Oases**																

***k_c***	**locus**	**max length**	**N50**	**>1-trans locus**	**max trans**	**avg trans**	**total hits**	**unique hits**	**>1-hit locus**	**max hits**	**time** **(mins)**			**memory** **(GB)**		

35_3	39584	15586	801	3824	13	3	29928	10898	256	4	94,28			29,32		
35_5	28537	15586	936	2616	16	3	22460	10103	245	4	94,26			29,30		
35_10	17075	11104	982	1377	14	3	13800	8201	185	5	94,24			29,26		

**Trans-ABySS**																

***k_c***	**trans**	**max length**	**N50**	**>1-node trans**	**max nodes**	**avg nodes**	**total hits**	**unique hits**	**time** **(mins)**				**memory** **(GB)**			

35_3	91365	15586	898	50467	60	8	33600	10639	205,1				4,1			
35_5	55164	10582	997	27763	46	7	25779	9944	195,1				4,1			
35_10	28455	8865	929	13665	43	6	16032	8154	178,1				4,1			

The *k*-mer length is fixed to 35 because Oases is only capable of assembling these libraries on machines with 32 GB physical memory when *k *is large. For our postprocessing algorithm, the notations are the same as in Table 1. For Oases, locus denotes the number of predicted locus, max length denotes the length of the longest predicted transcript, N50 denotes the N50 value of the longest transcript length in a predicted locus, >1-trans locus denotes the number of predicted locus with more than one transcript, max trans denotes the maximum number of transcripts in a predicted locus, avg trans denotes the average number of transcripts in predicted locus with more than one transcript, total hits denotes the total number of hits from translated BLAST search of each predicted transcript to *Drosophila *(isoforms are considered the same gene, only the top hit with *E*-value below 10^−^^7 ^is considered for each transcript in a predicted locus, and hits from transcripts within the same predicted locus to the same gene are counted once), unique hits denotes the number of unique hits to different genes, >1-hit locus denotes the number of predicted locus that has BLAST hits to more than one gene, max hits denotes the maximum number of different genes that have BLAST hits to a predicted locus, time (mins) denotes the computational time in minutes, with the values to the left and to the right of "," indicating the running time of Velvet (without setting cov_cutoff) and Oases respectively, and memory (GB) denotes the memory requirement in gigabytes, with the values to the left and to the right of "," indicating the memory requirement of Velvet (without setting cov_cutoff) and Oases respectively. For Trans-ABySS, trans denotes the total number of predicted transcripts, max length denotes the length of the longest predicted transcript, N50 denotes the N50 value of the length of predicted transcripts, >1-node trans denotes the number of predicted transcripts that are the concatenation of more than one node, max nodes denotes the maximum number of nodes in a predicted transcript, avg nodes denotes the average number of nodes in predicted transcripts with more than one node, total hits denotes the total number of predicted transcripts that have BLAST hits, unique hits denotes the number of unique hits to different genes, time (mins) denotes the computational time in minutes, with the values to the left and to the right of "," indicating the running time of ABySS and Trans-ABySS respectively, and memory (GB) denotes the memory requirement in gigabytes, with the values to the left and to the right of "," indicating the memory requirement of ABySS and Trans-ABySS respectively.

**Figure 7:**
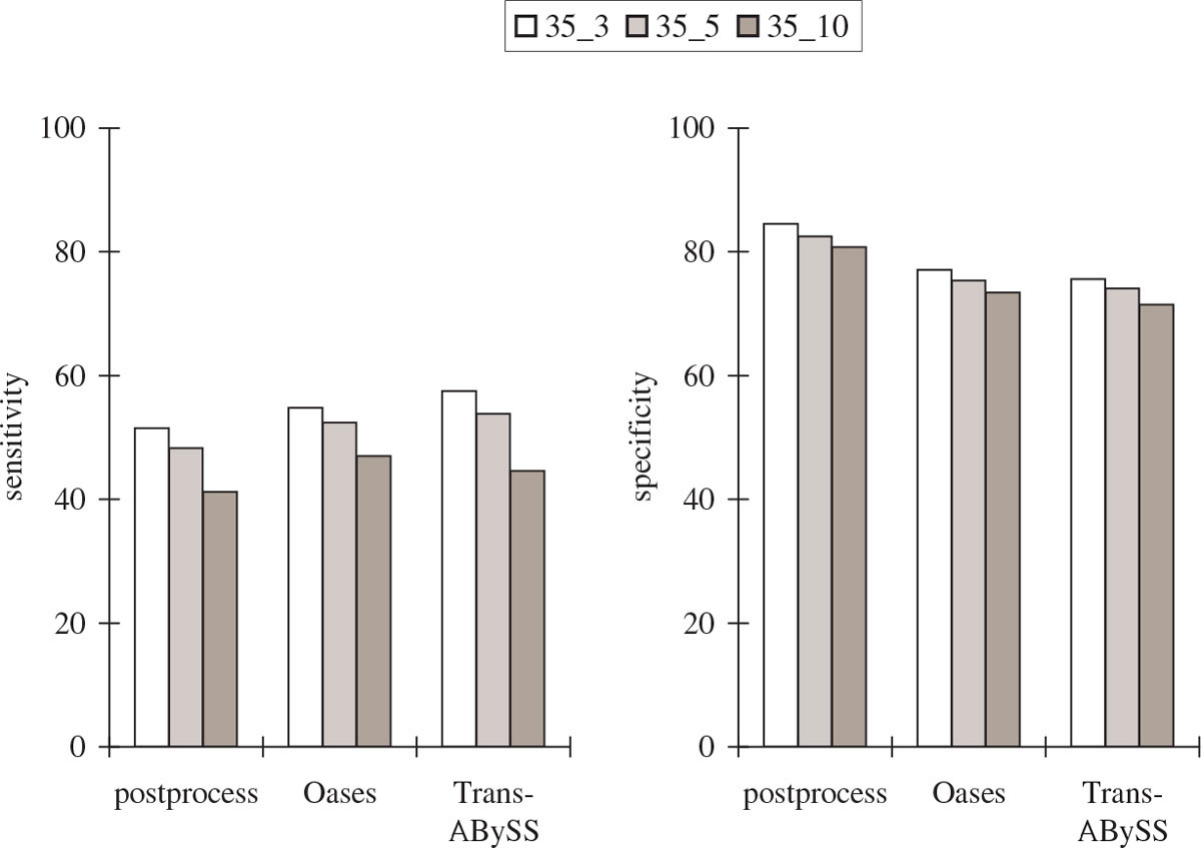
**Comparisons of the protein sequence BLAST results in the *Drosophila* transcriptome assemblies using six publicly available libraries with *k* = 35 and over different values of *k*-mer coverage cutoff *c* (represented by *k*_*c*)**. The notations are the same as in Figure 5.

**Figure 8:**
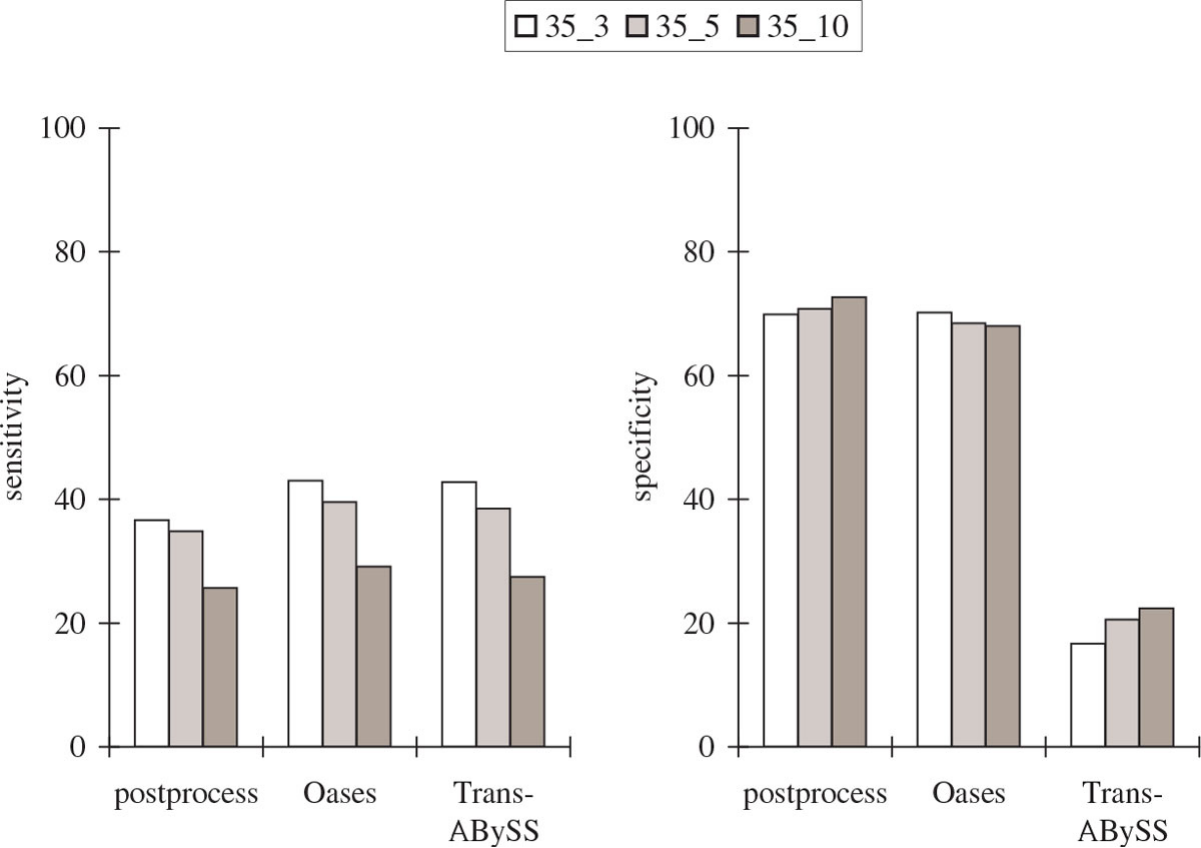
**Comparisons of the alternative splicing junction results in the *Drosophila* transcriptome assemblies using six publicly available libraries with *k *= 35 and over different values of *k*-mer coverage cutoff *c *(represented by *k_c*)**. The notations are the same as in Figure 6.

Figure 7 and Figure 8 show that although our postprocessing algorithm had the lowest sensitivity with respect to protein sequence BLAST and alternative splicing junctions, it had the highest sequence specificity. Both our postprocessing algorithm and Oases had the highest specificity with respect to alternative splicing junctions, although it was not as high when compared to the simulation results.

**Figure 9:**
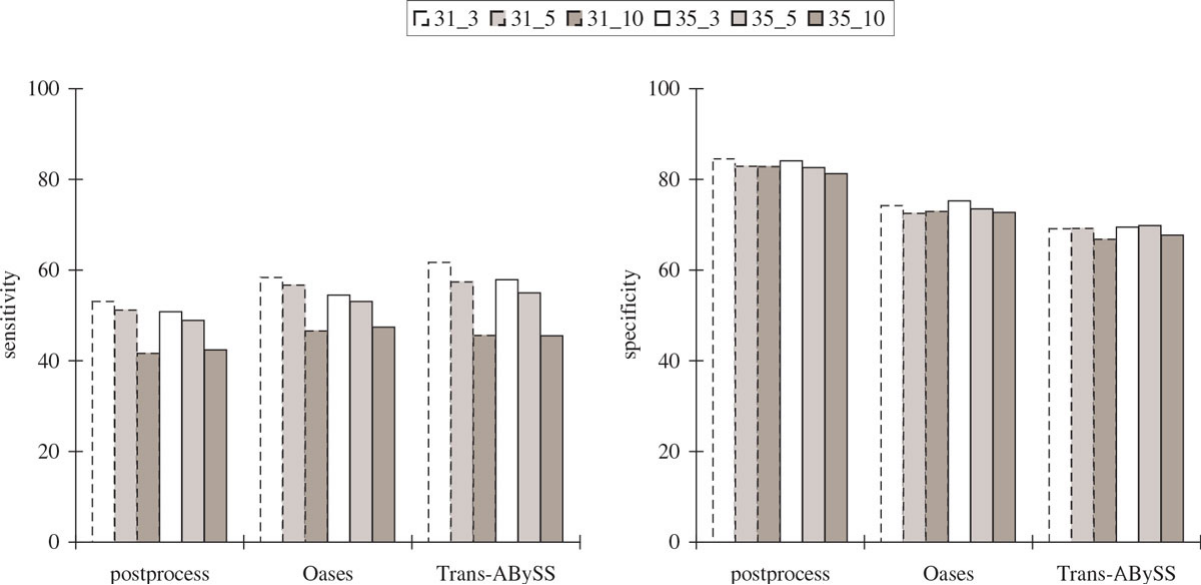
**Comparisons of the protein sequence BLAST results in the *Drosophila* transcriptome assemblies using four publicly available libraries over different values of *k *and *k*-mer coverage cutoff *c *(represented by *k_c*)**. The notations are the same as in Figure 5.

**Figure 10:**
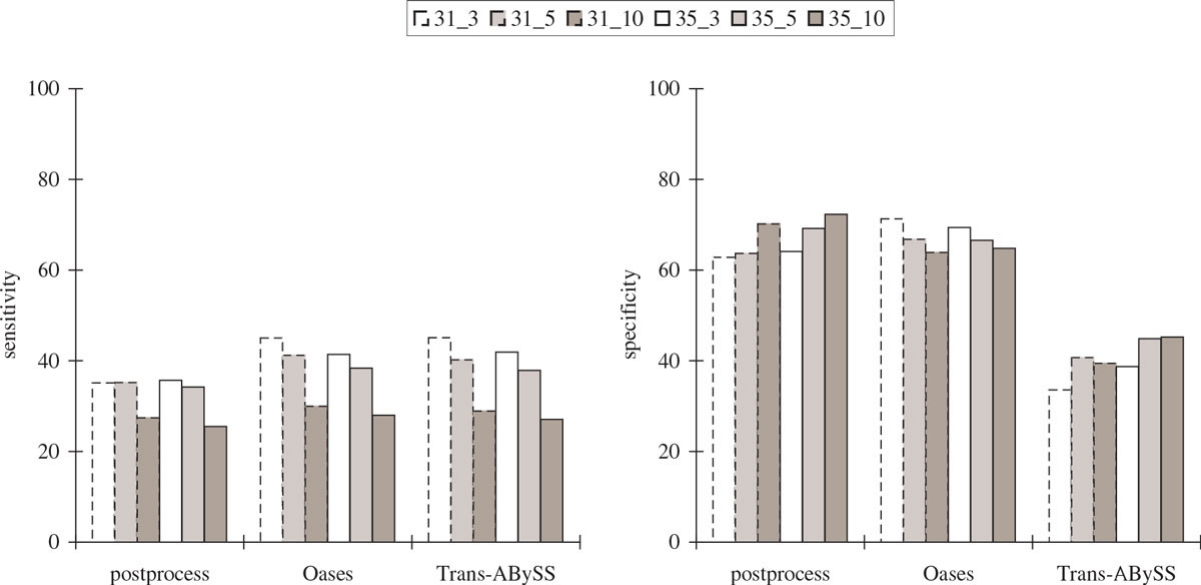
**Comparisons of the alternative splicing junction results in the *Drosophila* transcriptome assemblies using four publicly available libraries over different values of *k *and *k*-mer coverage cutoff *c *(represented by *k_c*)**. The notations are the same as in Figure 6.

In order to evaluate the performance with varying *k*, we also considered a smaller set of four libraries by removing the second replicate from the mixed pupal libraries and replacing the two adult libraries with a single adult library at a different time point (SRX019657), giving a total of 76819166 reads with average length 42 after quality trimming. Table 4, Figure 9 and Figure 10 show that the results had similar characteristics as the ones with six libraries, with better performance when *k *= 31.

**Table 4:** Comparisons of the *Drosophila* transcriptome assemblies of our postprocessing algorithm, Oases and Trans-ABySS using four publicly available libraries over different values of *k *and *k*-mer coverage cutoff *c*. The notations are the same as in Table 3.

postprocess																
***k*_c**	**initial** **nodes**	**largest tangle**	**largest SCC**	**splicing graphs**	**max length**	**N50**	**>1-node** **graphs**	**max node**	**avg nodes**	**SNPs**	**total hits**	**unique hits**	**>1-hit graphs**	**max hits**	**time** **(mins)**	**memory** **(GB)**

31_3	293034	251819	132958	87306	7571	542	1914	36	5	13216	37135	10752	516	7	81,24	20,2
31_5	153123	115511	60504	53199	9708	748	1881	98	5	8419	27103	9868	683	8	81,23	20,2
31_10	70809	36861	19839	35955	7393	621	1224	108	5	3746	22037	8399	442	8	81,21	20,2

35_3	175184	123605	85923	73584	7525	559	2246	79	6	10311	37115	10565	737	8	81,22	20,2
35_5	98897	58409	40689	47081	9382	731	1808	134	6	6741	26560	9631	743	12	81,21	20,2
35_10	48595	19438	13375	28269	7008	706	1062	90	5	2967	17829	7883	461	8	81,19	20,2

**Oases**																

***k*_c**	**locus**	**max length**	**N50**	**>1-trans locus**	**max trans**	**avg trans**	**total hits**	**unique hits**	**>1-hit locus**	**max hits**	**time** **(mins)**			**memory** **(GB)**		

31_3	35587	15986	994	4881	18	3	26559	10819	410	5	87,24			25,27		
31_5	26679	15906	1109	3234	20	3	21084	9944	336	5	87,21			25,21		
31_10	21283	8174	877	1637	16	3	17225	8449	188	4	87,19			25,21		

35_3	37377	9826	846	3724	16	3	28492	10652	346	6	75,14			17,17		
35_5	27573	12562	979	2644	14	3	21992	9751	332	5	75,13			17,17		
35_10	18072	7934	939	1389	12	3	14614	7953	194	5	75,12			17,17		

**Trans-ABySS**																

***k*_c**	**trans**	**max length**	**N50**	**>1-node trans**	**max nodes**	**avg nodes**	**total hits**	**unique hits**	**time** **(mins)**				**memory** **(GB)**			

31_3	113157	14353	1149	72266	56	6	33780	10527	201,1				4,1			
31_5	62292	14395	1282	37656	72	6	24614	9810	193,1				4,1			
31_10	32509	17057	1075	19837	50	5	16676	8313	172,1				4,1			

35_3	76220	14351	1142	40606	79	6	31619	10288	179,1				4,1			
35_5	46431	14385	1239	23632	38	5	23451	9603	172,1				4,1			
35_10	24956	9139	1095	12968	30	5	15057	7956	154,1				4,1			

## Conclusions

We have developed a postprocessing algorithm that can recover alternative splicing information directly from de Bruijn graphs of RNA-Seq data. Our strategy does not require prior genomic knowledge and supports the study of differential expression through investigating *de novo *RPKM values [11]. The computational time is linear in the size of the de Bruijn graph, and our algorithm takes a few minutes to half-an-hour to complete after results from Velvet are available in the test cases (see Tables 1-4). It uses less memory than Velvet, while running Oases together with Velvet without setting cov_cutoff is often more memory-intensive than running Velvet with cov_cutoff (see Tables 1-4). Our algorithm performs well on simulations with low percentages of mismatches in the reads and generally has higher specificity than Oases or Trans-ABySS. It is most suitable in situations in which a more reliable assembly is desired at the expense of lower sensitivity. Our algorithm has been applied to perform transcriptome assembly of the non-model blow fly *Lucilia sericata *in [11], which allows comparison of its transcriptome to the closely related model organism *Drosophila *through translated BLAST search, investigation of alternative splicing and differential expression among various developmental stages, and identification of transposable elements.

## Competing interests

The authors declare that they have no competing interests.

## Authors' contributions

AMT and S-HS designed the computational work, analyzed the data and wrote the paper. S-HS did the simulation experiments. All authors read and approved the final manuscript.

## Declarations

Publication costs for this work were funded by the Open Access to Knowledge (OAK) Fund at the Texas A&M University Libraries.

This article has been published as part of *BMC Genomics *Volume 15 Supplement 5, 2014: Selected articles from the Third IEEE International Conference on Computational Advances in Bio and Medical Sciences (ICCABS 2013): Genomics. The full contents of the supplement are available online at http://www.biomedcentral.com/bmcgenomics/supplements/15/S5.
